# Ethical Decision-Making for Self-Driving Vehicles: A Proposed Model & List of Value-Laden Terms that Warrant (Technical) Specification

**DOI:** 10.1007/s11948-024-00513-0

**Published:** 2024-10-10

**Authors:** Franziska Poszler, Maximilian Geisslinger, Christoph Lütge

**Affiliations:** 1 Peter Löscher Chair of Business Ethics, Institute for Ethics in Artificial Intelligence, Technical University of Munich (TUM), Arcisstraße 21, 80333 Munich, Germany; 2 Institute of Automotive Technology, Technical University of Munich (TUM), Boltzmannstr. 15, 85748 Garching b. Munich, Germany

**Keywords:** Self-driving vehicle, Autonomous driving, Ethical decision-making, Computational ethics, Risk distributions, Risk ethics

## Abstract

Self-driving vehicles (SDVs) will need to make decisions that carry ethical dimensions and are of normative significance. For example, by choosing a specific trajectory, they determine how risks are distributed among traffic participants. Accordingly, policymakers, standardization organizations and scholars have conceptualized what (shall) constitute(s) ethical decision-making for SDVs. Eventually, these conceptualizations must be converted into specific system requirements to ensure proper technical implementation. Therefore, this article aims to translate critical requirements recently formulated in scholarly work, existing standards, regulatory drafts and guidelines into an explicit five-step ethical decision model for SDVs during hazardous situations. This model states a precise sequence of steps, indicates the guiding ethical principles that inform each step and points out a list of terms that demand further investigation and technical specification. By integrating ethical, legal and engineering considerations, we aim to contribute to the scholarly debate on computational ethics (particularly in autonomous driving) while offering practitioners in the automotive sector a decision-making process for SDVs that is technically viable, legally permissible, ethically grounded and adaptable to societal values. In the future, assessing the actual impact, effectiveness and admissibility of implementing the here sketched theories, terms and the overall decision process requires an empirical evaluation and testing of the overall decision-making model.

## Introduction

Self-driving vehicles (SDVs) are one of the first commercialized AI-enabled robots to make decisions without human intervention. Although greater safety levels are attributed to SDVs than human drivers, they may end up in situations with fatal consequences for traffic participants (Rhim et al., 2020). While (ethical) decision-making of human drivers in traffic is rather intuitive, SDV’s decision-making must be preprogrammed (Liu & Liu, 2021). Some opponents may argue that ethical computing (for dilemma situations) is irrelevant (UNECE, 2021); others point to its inevitability: “Even in instances in which no explicit ethical rule or preference is intended, the programming of a [SDV] may establish an implicit or inherent decision rule with significant ethical consequences” (U.S. Department of Transportation, 2016, p. 26). For example, while automotive companies rather speak of risk management of SDVs and refrain from using the term ‘ethics’, their (implicit) programming and management of risks have palpable effects on all road users in terms of risk impositions (Evans, 2021) or—in the worst case—traffic victims (Mordue et al., 2020). For instance, in 2014, Google released a patent that focused merely on positioning SDVs in a way that minimizes the risk exposure of passengers (Dolgov & Urmson, 2014), while neglecting the risk levels of vulnerable road users outside the vehicle, which can have devastating consequences for these types of road users (Geisslinger et al., 2021). In later Google patents (e.g., Teller & Lombrozo, 2023), also pedestrians and their respective risk magnitudes are considered. In any case, such SDV’s decisions (i.e., trajectory selection) are not solely objective assessments and managements of risks but carry an ethical dimension and are of normative significance (Dietrich & Weisswange, 2019; Taebi, 2021).

Considering the risks at hand and the fast-paced technological advancements, investigating the ethical programming of SDVs is a pressing concern (Nyholm & Smids, 2016), with the objective of potentially establishing a standardized, mandatory framework for such programming that serves the interests of society (Gogoll & Müller, 2017). Thus, many scholars have discussed what should feed into the programming and what constitutes ethical decision-making for SDVs. For example, to approach this conceptualization, Poszler et al. (2023) have conducted a holistic review of the autonomous driving ethics literature, in which they evaluated the applicability of certain ethical theories, identified additional considerations (such as situation-adjusted risk distributions) that may prove helpful to guide SDVs’ ethical decision-making and synthesized ethical trajectory-planning algorithms that have been developed so far. Similarly, policymakers have recognized the importance of considering ethical dimensions in programming SDVs (e.g., European Commission, 2021; U.S. Department of Transportation, 2016). In this endeavor, compared to ‘meaningless’ and ‘toothless’ principles that should underlie AI development processes, such as fairness and privacy, operationalizing certain principles directly into product features seems to be a more promising approach (Munn, 2022). In the future, eliciting ethical values and turning them into concrete system requirements will be a regular task for system designers and so-called ‘value leads’ (compare IEEE 7000; IEEE, 2021). However, in terms of SDVs, “translating rules of the road, which are [amongst others] legal documents written in natural language, to formal rules for use by computers deployed on [autonomous vehicles] is a challenging task” (Bin-Nun et al., 2022, p. 1).

Therefore, this article’s objective is to support this endeavor by translating requirements recently formulated in policy drafts, standards, technical specifications, ethics guidelines and scholarly work into an explicit five-step ethical decision model for SDVs during hazardous situations. We, hereby, aim to extend previous efforts by merging three key aspects: ethical, legal and engineering considerations. For example, former scholarly work “rather focused on blending normative and empirical accounts of what matters morally in AV [i.e., autonomous vehicle] decision-making” (Evans, 2021, p. 326) while transgressing restrictions that today have been legally specified, such as the discrimination of traffic participants based on personal features (e.g., age) (BMJ, 2023). Similarly, it has been suggested that engineering standards “may be reconsidered for future revision, including normative requirements” (SAE International, 2021a). With our simultaneous consideration of ethics, law and engineering, we hope to achieve a higher level of social acceptance, ethical acceptability, legal substantiation and technical feasibility of the resulting decision-making model for SDVs.

This paper is structured as follows. First, “Theoretical Fundamentals” section highlights theoretical fundamentals drawn from policymakers, standardization organizations and contemporary scholars. Second, “A Proposed Model for Ethical Decision-Making of SDVs” section sketches the proposed model for the ethical decision-making of SDVs and elaborates its decision process with an exemplary traffic scenario. Third, the benefits and limitations of this model are illustrated and underlying terms that need concretization in the future are pointed out. Lastly, a short conclusion is drawn. Overall, although not exhaustive and resolute, this article aims to serve the scholarly community by contributing to the debate on computational ethics and value-based engineering (especially in the field of autonomous driving) and practitioners in the automotive sector by laying out a potential solution for the ‘ethical’ programming of SDVs.

## Theoretical Fundamentals

This section provides a short overview of premises and requirements for an SDV’s ethical decision-making that were raised by policymakers and scholars in the past and represents the fundament on which the subsequent proposed model is built. Overall, these requirements are fourfold: the management of risks (i.e., safety) (“Risk (i.e., Safety) Assessment and Management” section.), adjustments to the underlying calculation depending on the situation criticality (“Adjustments to the Underlying Calculation Depending on the Situation's Criticality” section.), responsibility considerations and the protection of vulnerable road users (“Responsibility Considerations and the Protection of Vulnerable Road Users” section.) as well as implementing a mix of ethically grounded and socially shared principles (“Implementing a Mix of Ethically Grounded and Socially Shared Principles” section.).

### Risk (i.e., Safety) Assessment and Management

Generally, regulatory frameworks for AI systems point to assessing and managing pertinent applications using risk-based approaches (e.g., European Commission, 2021). The consideration of risks similarly exhibits centrality in regulatory drafts that specifically concern the functionality and programming of SDVs. In this context, risk is specified as the “combination of the probability of occurrence of harm and the severity of that harm” (ISO, 2018b) or the product of collision probability and estimated harm (Geisslinger et al., 2021). Especially in the sight of and during critical traffic scenarios (defined in “Responsibility Considerations and the Protection of Vulnerable Road Users” section.), SDVs are expected to calculate the probability and magnitude of imminent consequences (e.g., Dignum, 2019) and minimize risks to the safety of traffic participants by executing a maneuvering to reach a ‘minimal risk condition’ (e.g., European Commission, 2022; Justia US law, 2022). This condition is characterized by ensuring the greatest possible road safety for all road users (e.g., ISO TC 241; BMJ, 2023). Safety is understood in terms of physical integrity in that the protection of human life and reduction of road fatalities is given the highest priority over other considerations such as damage to property (e.g., BMJ, 2023; Kriebitz et al., 2022; Lütge et al., 2021). Furthermore, the safety/protection of passengers within the SDV must not be prioritized over third parties within the traffic scenario. Instead, even consideration of all road users is postulated (e.g., European Commission, 2022; Government of Canada, 2021; GOV.UK, 2023; NACTO, 2016). This commitment to foregoing risk inequality is further manifested by the demand for SDVs to “contribute to the reduction of the disproportional risk exhibited by certain road user groups” (Papadimitriou et al., 2022, p. 3), which will be further elaborated in “Responsibility Considerations and the Protection of Vulnerable Road Users” section. Key requirements for SDVs can be summarized as follows:

**Table:** 

System requirements	Corresponding standards & regulations (Examples & further readings^a^)
Calculation of risks (i.e., product of collision probability and estimated harm)	Definition of ‘risk’ in the context of SDVs (ISO, 2018b)
Performance of maneuvers to reach a ‘minimal risk condition’	Constraints to avoid collisions with all other objects and to maintain a safe distance from other objects (GOV.UK, 2022) Definition of ‘minimal risk condition’ and ‘minimal risk manoeuvre’ (DMV, 2022; European Commission, 2022; Government of Canada, 2021; ISO, 2021b; ISO, 2022; Justia US law, 2022; Ministères Écologie Énergie Territoires, 2022; SAE International, 2021b; U.S. Department of Transportation, 2016)
Minimal risk = greatest possible safety (i.e., physical integrity of human beings)	Definition of ‘minimal risk’ (ISO TC 241; BMJ, 2023) Definition of ‘safety’ in the context of SDVs (i.e., in terms of physical integrity and road fatalities) (BMJ, 2023)
Simultaneous consideration of the safety of traffic participants in and outside the SDVs	Consideration of all road users beyond passengers (i.e., third parties, the broader transport ecosystem) (BMJ, 2023; European Commission, 2022; Government of Canada, 2021; GOV.UK, 2023; NACTO, 2016; UNECE, 2022)

Exemplary references to standards and regulations that address the ‘ethical’ decision-making of SDVs, while general regulations or standards that did not concern SDVs’ programming were excluded, such as the U.S. Department of Transportation (2021). All standards and regulations consulted in this article are listed in the Appendix. This list should not be understood as an exhaustive compilation but as a guide outlining some key aspects of existing law and established standards

### Adjustments to the Underlying Calculation Depending on the Situation’s Criticality

In general, SDVs are expected to increase road safety (Lütge, 2017) and, thus, mostly encounter collision-free situations. Nevertheless, not all risks can be bypassed by the introduction and operation of SDVs (Goodall, 2016), so collisions may emerge. Correspondingly, this duality of traffic scenarios has been recognized by introducing terms such as ‘non-hazard’ and ‘hazard’ situations (Dietrich & Weisswange, 2019) or ‘non-critical’ and ‘critical’ occurrences (UNECE, 2022). In contrast to ‘non-critical’ situations, ‘critical situations’ entail that “at least one person suffers an injury that requires medical attention” (Ministères Écologie Énergie Territoires, 2022; UNECE, 2022, p. 32). The distinction between these types can be based on certain metrics and risk thresholds and be followed by a remedial action in case an unacceptable risk is predicted (UNECE, 2022). Such an ad hoc action in ‘critical’ situations is characterized by selecting the trajectory that protects human life above everything else, such as damages to animals or property (Zhu, 2021) or strict compliance with road traffic laws (e.g., crossing a solid line to pass a cyclist) (NTC, 2022). Therefore, human life (i.e., physical integrity) is the only factor entering the outcome calculation when comparing different trajectories. On the other hand, in ‘non-critical’ situations, additional factors can be consulted when determining the optimal trajectory, such as mobility or passenger comfort (e.g., Dietrich & Weisswange, 2019; Geisslinger et al., 2023a; Westhofen et al., 2023). Thus, the categorization of traffic situations is decisive for the data consulted in the SDV’s calculation and decision-making process. Key system requirements can be summarized as follows:

**Table:** 

System requirements	Corresponding standards & regulations (Examples & further readings)
Separation of traffic situations into ‘hazard’ and ‘non-hazard’ and corresponding SDV responses	Distinction of situations into ‘non-critical’ and ‘critical’ occurrences or approaches (ISO, 2023a; UNECE, 2022) or ‘normal driving’ and ‘crash avoidance’ responses (U.S. Department of Transportation, 2016) Definition of ‘dilemmas’ as critical situations (European Commission, 2020) Definition of ‘critical situations’ and ‘personal road traffic accidents” (European Commission, 2022; Ministères Écologie Énergie Territoires, 2022; UNECE, 2022) Definition of ‘hazardous situation’ (IEEE, 2022; ISO, 2020a) Definition of ‘conflict zone’ (ETSI, 2018)
Criticality determination based on metrics, thresholds relating to unacceptable risk	Emphasis of minimum threshold level of safety & unacceptable risk (Government of Canada, 2021; UNECE, 2022) Unacceptable risk depends on factors such as the level of controllability from other road users, rates of occurrence or severity levels of the outcome of a particular traffic scenario (IEEE, 2022) Predefined collision risk probability determines the need to initiate actions to avoid a collision (ETSI, 2018, 2021b) Indicators for the criticality of the traffic safety situation, such as Time-to-collision or the time required to act to avoid or mitigate a collision (ETSI, 2018, 2021b; ISO, 2021a, 2023a)
Consideration of a single parameter (i.e., human life) in ‘hazard’ situations	Protection of human life as the highest priority compared to other legal interests (BMJ, 2023) Primary road safety application is to prevent collisions (ETSI, 2018, 2021b) Examples of obligations that can be neglected, e.g., strict compliance with road traffic law (NTC, 2022)

### Responsibility Considerations and the Protection of Vulnerable Road Users

As stated in “Risk (i.e., Safety) Assessment and Management” section., risk distributions are, in principle, to be allocated equally between all traffic participants. Berkey (2022) endorses this with a side constraint, namely, “unless there is a morally compelling reason for deviating from this aim” (p. 11), such as allocating a greater share of risks to those road users who introduce risks in the first place. This aligns with the concept of moral responsibility, which holds that drivers, despite any precautions, are responsible for causing harm in the event of a collision since they voluntarily engage in activities that threaten others (Kauppinen, 2021). By contrast, road users such as cyclists or pedestrians impose much less risk on road traffic due to their mass and velocities (Geisslinger et al., 2023a). Therefore, although owners of SDVs may not be liable for causing an accident (Jensen, 2018), SDVs could be programmed to assume higher levels of risk compared to other road users in critical situations. In practice, a vulnerability categorization of the road users is proposed, in which cyclists, pedestrians or generally, individuals outside the vehicle are distinguished from individuals inside the SDV (Evans et al., 2020). The necessity to protect vulnerable road users and, hence, balance risks between different classes of road users is similarly acknowledged by regulatory bodies (e.g., European Commission, 2022; Government of Canada, 2021; GOV.UK, 2022/2023; NHTSA, 2022). Key requirements can be summarized as follows:

**Table:** 

System requirements	Corresponding standards & regulations (Examples & further readings)
In principle, equal treatment of all traffic participants	Equality in the safety of all road users (European Commission, 2020)
Special protection for vulnerable traffic participants	Commensurate level of safety for vulnerable road users (European Commission, 2020; GOV.UK, 2022; ISO, 2023b; NHTSA, 2022)
Categorization of road users in line with their relative vulnerability	Definition of ‘vulnerable’ and ‘non-vulnerable’ road users (ISO, 2020b) Definition and characterization of ‘vulnerable road users’ in terms of parameters such as speed and weight class (ETSI, 2021b) Definition of ‘heavy vehicles’ (ISO, 2018a)
Potential categorization into vehicles, cyclists, pedestrians or users inside and outside an SDV	List/examples of ‘vulnerable road users’ (ETSI, 2018/2021; European Commission, 2020/2022; Government of Canada, 2021; IEEE, 2022; NHTSA, 2022)

### Implementing a Mix of Ethically Grounded and Socially Shared Principles

To achieve ethical decision-making and behavior of autonomous systems, it is important to integrate society’s values and ethical principles within the underlying algorithms (Dyoub et al., 2020). Ethical principles are here understood as “[o]perationalizable rules inferred from philosophical theories such as Deontology and Consequentialism” that help determine the moral permissibility of an action (Woodgate & Ajmeri, 2022, p. 3). Next to these normative theories, societal preferences can inform the decision-making logic of SDVs (Poszler et al., 2023). Specifically for SDVs, scholars highlight the need to create ‘mixed’ algorithms that combine various normative ethical theories (Geisslinger et al., 2021; Hübner & White, 2018) and society’s values (Robinson et al., 2021). For example, Evans et al. (2023) propose using Kantian, Millian or descriptive ethics as sources of inspiration for restrictions and mitigation rules that can be integrated into the ‘soul’ (i.e., the algorithm) of SDVs. This allows accounting “for a variety of ethical concerns [a]nd […] achieve widespread acceptance in society” (Evans, 2021, p. 324).

Similar to other fields in applied ethics, such as the ethics of radiation protection (Hansson, 2007), a mix of normative ethical theories is also proposed when it comes to SDVs. This way, unfair outcomes of relying on a single theory may be mitigated by utilizing a ‘balanced approach’ (ISO, 2023b) or ‘pluralistic approach’, in which “a variety of principles can be weighed against one another in order to find the fairest answer” (Woodgate & Ajmeri, 2022, p. 12). For example, there may be instances where it becomes unreasonable to follow strict duties, such as prioritizing the absolute protection of bystanders such as pedestrians (as indicated as a requirement in “Responsibility Considerations and the Protection of Vulnerable Road Users” section.). Such a notion of reasonableness could allow accepting minimal chances of harming an individual if, as a result, another person is saved with certainty (Sütfeld et al., 2019). Similarly, it might be justifiable to deviate from aiming at an equal distribution of the risks (as indicated as a requirement in “Responsibility Considerations and the Protection of Vulnerable Road Users” section.) to reduce the absolute level of risk (i.e., harm) that every traffic participant is subjected to Berkey (2022). Naturally, conflicts and inconsistencies will emerge when consulting various normative theories simultaneously. For example, in the previously mentioned case, the level of risk imposition to one individual may compete with the aggregated level of risk for all traffic participants. When such conflicts emerge, humans can resolve these situations by accepting tradeoffs, developing hierarchical relations or assigning weights (IEEE, 2019). Therefore, regulatory bodies emphasize managing the decision-making of SDVs by “shared ethical principles” that align with societal values and preferences (European Commission, 2020, p.7). However, preferences indicated in empirical studies cannot be blindly adopted due to “the risk of committing the naturalistic fallacy” (Jacobs & Huldtgren, 2021, p.24). For example, in the Moral Machine experiment, participants stated they preferred sacrificing people who are old, overweight or homeless in accident situations involving SDVs (Awad et al., 2018). Even if this data could be estimated using sensor measurements (Németh, 2023), the European Commission (2020) prohibits the discrimination of humans based on their personal characteristics in critical situations. Thus, a promising approach seems to complement normative theories with societal values that comply with regulations (Dignum, 2019). Key requirements can be summarized as follows:

**Table:** 

System requirements	Corresponding standards & regulations (Examples & further readings)
Integration of a mix of normative theories, such as deontological and consequentialist ethics	Balanced approach that emphasizes the consideration of different normative ethical theories (ISO, 2023b)
Consideration of the reasonableness of risk impositions	Freedom of unreasonable safety risks (European Commission, 2022; ISO, 2022; NHTSA, 2022; UNECE, 2022; U.S. Department of Transportation, 2016) Safety = absence of unreasonable risk (ISO, 2020b; ISO, 2018b)
Alignment with society’s values via manifesting their preferred hierarchical orders, thresholds or weights	Importance of ‘shared’ ethical principles when managing risk distributions (European Commission, 2020) Acceptance criterion that derives unreasonable levels of risk from ‘valid societal moral concepts’ (ISO, 2022)
Prohibition to discriminate based on personal characteristics of humans	Risk distributions based on personal characteristics are forbidden (BMJ, 2023) Risk calculations should instead be based on physical properties such as the dynamic state and mass of the objects (ETSI, 2021b)

## A Proposed Model for Ethical Decision-Making of SDVs

Based on the groundwork stated in the previous section, this paper proposes a five-step model for ethical decision-making of SDVs and elaborates its decision process with an exemplary, simplified traffic scenario. The overall sequence of steps is illustrated in Fig. 2. All relevant terms and examples of its technical measures/indicators are defined in Table 9.

*Step 1: Determination & calculation of possible trajectories.* Decisions of SDVs are implemented via trajectory planning and selection. Thus, in the first step, the SDV needs to determine all potential trajectories and calculate corresponding consequences for each trajectory alternative ($A_{i}$) and each traffic participant ($T_{i}$). Consequences that play a role in road traffic include, first and foremost, safety (i.e., the physical integrity of the traffic participants, determined by the risk posed to them). The risk for each traffic participant ($r_{Ai,Ti}$) can be defined as the product of collision probability ($c_{Ai,Ti}$) and estimated harm $(h_{Ai,Ti}$). Additional—yet subordinated—utilities or objectives ($x_{Ai,Ti}$) of SDVs include passengers’ comfort or mobility. The calculation that is necessary for this step is grasped by Eq. 1:^1^

1 $$r_{Ai,Ti} = c_{Ai,Ti} h_{Ai,Ti}$$

**Fig. 1:**
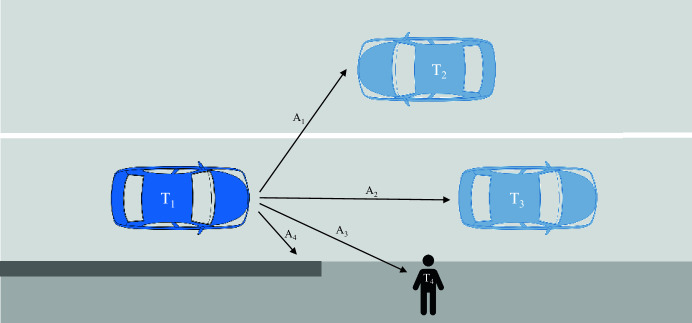
Simplified traffic scenario

***Exemplary elaboration****. In the imagined traffic scenario (*Fig. 1*), the SDV* ($T_{1}$) *has four trajectory alternatives, which are:* ($A_{1}$) *collide with an oncoming vehicle* ($T_{2}$) *to the left,* ($A_{2}$) *collide with a vehicle* ($T_{3}$) *in front,* ($A_{3}$) *collide with a pedestrian* ($T_{4}$) *on the sidewalk or* ($A_{4}$) *crash into a wall.*^2^

*For each of the four trajectories, the SDV calculates consequences, for example, the risk for each traffic participant (*$r_{Ai,Ti}$*) and any additional relevant utilities/objectives (*$x_{Ai,Ti}$*), as illustrated in *Table 1*. In this example, the numerical figures for risk range from 0 (i.e., no collision, entailing no injury) to 1 (i.e., certain collision, entailing a fatal injury). Similarly, the numerical figures for* $x_{Ai,Ti}$ *range from 0 (e.g., no comfort) to 1 (e.g., complete comfort).*

**Table 1:** Overview of risks ($r_{Ai,Ti}$) and for an unspecified utility/objective ($x_{Ai,Ti}$) for all trajectory alternatives and the individual traffic participants

Trajectory alternatives (${A}_{i}$)	Traffic participants (${T}_{i}$)								
	${T}_{1}$			${T}_{2}$		${T}_{3}$		${T}_{4}$	
	${c}_{Ai,T1}$	${h}_{Ai,T1}$	${x}_{Ai,T1}$	${c}_{Ai,T2}$	${h}_{Ai,T2}$	${c}_{Ai,T3}$	${h}_{Ai,T3}$	${c}_{Ai,T4}$	${h}_{Ai,T4}$
	${r}_{Ai,T1}$			${r}_{Ai,T2}$		${r}_{Ai,T3}$		${r}_{Ai,T4}$	
${A}_{1}$	0.50	0.50	0.5	0.60	0.40	0.00	0.00	0.00	0.00
	0.25			0.24		0.00		0.00	
${A}_{2}$	0.50	0.70	0.9	0.10	0.20	0.50	0.50	0.00	0.00
	0.35			0.02		0.25		0.00	
${A}_{3}$	0.90	0.20	0.8	0.00	0.00	0.00	0.00	0.80	0.90
	0.18			0.00		0.00		0.72	
${A}_{4}$	0.90	0.60	0.4	0.00	0.00	0.00	0.00	0.10	0.40
	0.54			0.00		0.00		0.04	

Strictly speaking, the collision probabilities for the traffic participants that are potentially colliding with each other are symmetrical. To elaborate, for trajectory $${A}_{3}$$, where the SDV ($${T}_{1})$$ potentially collides with the pedestrian ($$T_{4}$$), their collision probabilities should be equal. However, Table 1 presents varying collision probabilities for these two colliding parties (i.e., $$c_{Ai,T1}$$ = 0.9 and $$c_{Ai,T4}$$ = 0.8). This discrepancy is due to mutual influences among all traffic participants and other existing obstacles that we assume to know in this article.

*Step 2: Typification of situation.* The SDV determines the nature of the situation based on its ability to fulfill particular key duties. As the prime requirement for SDVs is safety, key duties (to be prioritized over utilities or objectives such as comfort or mobility) entail safeguarding the physical integrity of all traffic participants. Exemplary rules/duties are displayed in Table 2, which will be consulted when assessing traffic situations and consequences such as those illustrated in Table 1. If the SDV concludes that at least one of the established rules/duties will be disobeyed (determined by the surpassing of a particular risk figure), the SDV will declare a ‘hazard situation mode’ implicating a specific decision-making process (that is different from the ‘non-hazard situation mode’^3^). Namely, in the ‘hazard situation mode’, the only consequence to be contemplated is risk, while other consequences, such as the passenger’s comfort or mobility, are to be neglected.

**Table 2:** Exemplary duties for SDVs that determine non-hazard vs. hazard situation mode (adjusted from Evans et al., 2020)

If any of the following **duties** cannot be fulfilled, the SDV declares **‘hazard situation’** mode:	
1.	The lives of other traffic participants must not be put in harm’s way. (e.g., $r_{Ai,T2}$ must not > 0.2)^a^
2.	The lives of SDV passengers must not be put in harm’s way. (e.g., $r_{Ai,T1}$ must not > 0.2)

^a^The indicated numerical figures throughout this section are not to be taken at face value; they are only stated as examples for explanatory purposes. In reality, to compete with human driving performance, risks must be much smaller, e.g., the EU legislation uses a value of 10^–7^ fatalities per hour as an example. The discussion section of this article highlights how to approximate the actual numerical figures that are to be utilized as reference points in practice

***Exemplary elaboration****. In the imagined situation (*Fig. 1*), all four trajectory alternatives entail risk above the level of 0.2 for each traffic participant (as illustrated in *Table 1*). According to the commands in *Table 2*, this means the SDV cannot fulfill its duty to not cause harm to traffic participants and/or the passenger(s) of the SDV. Therefore, the SDV will switch to ‘hazard situation mode’, implicating that utilities/objectives such as comfort or mobility are no longer directive for the subsequent calculations.*

*Step 3: Exclusion of prohibited trajectories.* To identify prohibited trajectories, the SDV checks the consequences of all trajectory alternatives for every traffic participant against particular risk thresholds. Thresholds to be contemplated are those for collision probability and estimated harm, each separately (i.e., $c_{max}$ and $h_{max}$). As indicated in Table 3, if the collision probability exceeds a particular numerical figure for one traffic participant, this individual’s estimated harm must not exceed a particular threshold. A similar logic applies if the estimated harm for a traffic participant exceeds a certain numerical figure. Those trajectories that fail to fulfill particular threshold restrictions are to be excluded; all remaining trajectory alternatives are reevaluated by the SDV’s algorithm in step 4.

**Table 3:** Exemplary threshold restrictions for collision probability (${\text{c}}_{{{\text{max}}}}$) and estimated harm (${\text{h}}_{{{\text{max}}}}$)

If $c_{Ai,Ti}$ > 0.8, $h_{Ai,Ti }$ must not > $h_{max}$ = 0.7 OR
If $h_{Ai,Ti}$ > 0.8, $c_{Ai,Ti}$ must not > $c_{max}$ = 0.6

***Exemplary elaboration****. According to the calculated consequences in *Table 1* (i.e.,* $c_{A3,T4}$ = *0.80 and* $h_{A3,T4}$ = *0.90) and the threshold restrictions in *Table 3*, the SDV has to exclude the trajectory alternative* $A_{3}$ *since for traffic participant* $T_{4}$ *none of the two restrictions is adhered to. Therefore, the SDV’s new action space for further consideration is limited to trajectory alternatives* $A_{1}$*,* $A_{2}$ *and* $A_{4}$*.*

*Step 4: Calculation of valence-adjusted risk.* The SDV reevaluates all remaining trajectory alternatives by adjusting the risk figures with valence factors ($$v_{i} )$$ for the different traffic participants. This valence factor corresponds to the traffic participant’s vulnerability. For example, traffic participants could be classified into pedestrians, cyclists and vehicles, with gradually declining valence factors, as illustrated in Table 4. The calculation that is necessary for this step is grasped by Eq. 2:

2 $$vr_{Ai,Ti} = v_{i} r_{Ai,Ti}$$

**Table 4:** Exemplary hierarchy and corresponding valence factors *(*${\text{v}}_{{\text{i}}} ){ }$ for different types of traffic participants

For pedestrians, $$v_{ped}$$ = 1.0
For cyclists, $$v_{cyc}$$ = 0.8
For motor vehicles, $v_{mot}$ = 0.5

The new valence-adjusted risk figures (i.e., $vr_{Ai,Ti}$) (illustrated in Table 5) feed into the decision-making process of Step 5 (Table 6).

**Table 5:** Overview of calculated valence-adjusted risks ($vr_{Ai,Ti}$) for all remaining trajectory alternatives and the individual traffic participants

Trajectory alternatives ($A_{i}$)	Traffic participants ($T_{i}$)											
	$T_{1}$			$T_{2}$			$T_{3}$			$T_{4}$		
	$r_{Ai,T1}$	$v_{mot}$	$vr_{Ai,T1}$	$r_{Ai,T2}$	$v_{mot}$	$vr_{Ai,T2}$	$r_{Ai,T3}$	$v_{mot}$	$vr_{Ai,T3}$	$r_{Ai,T4}$	$v_{ped}$	$vr_{Ai,T4}$
$A_{1}$	0.25	0.50	0.13	0.24	0.50	0.12	0.00	0.50	0.00	0.00	1.00	0.00
A_2_	0.35	0.50	0.18	0.02	0.50	0.01	0.25	0.50	0.13	0.00	1.00	0.00
$A_{4}$	0.54	0.50	0.27	0.00	0.50	0.00	0.00	0.50	0.00	0.04	1.00	0.04

**Table 6:** Exemplary weighting factors for risk inequality ($w_{E}$) and aggregated risk ($w_{U}$)

$$w_{E}$$ = 0.5
$$w_{U}$$ = 0.5

***Exemplary elaboration****. In the imagined, simplified scenario (*Fig. 1*), there are only two types of traffic participants, i.e., one pedestrian and motor vehicles. Therefore, utilizing the predetermined valence factors in *Table 4* (i.e.,* $v_{ped}$ *and* $v_{mot}$*), the SDV calculates the valence-adjusted risk (*$vr_{Ai,Ti}$*) for each traffic participant in each of the three remaining trajectory alternatives* $A_{1}$*,* $A_{2}$ *and* $A_{4}$*. This implies that the new risk figures for the involved vehicles (i.e.,* $vr_{Ai,T1}$*,* $vr_{Ai,T2}$*,* $vr_{Ai,T3}$*) are lower than their initially calculated risk (i.e.,* $r_{Ai,T1}$*,* $r_{Ai,T2}$*,* $r_{Ai,T3}$*). In contrast, the new risk figures for the involved pedestrian (i.e.,* $vr_{Ai,T4}$*) are not discounted but remain the same due to the valence factor for pedestrians of 1.0.*

*Step 5: Selection of final trajectory.* This step aims to identify the one trajectory that meets two risk distribution principles, namely, the greatest equal risk between traffic participants and that optimizes (i.e., minimizes) aggregated risk. Thus, based on the valence-adjusted risks, the SDV calculates the risk inequality ($E_{Ai}$) between all traffic participants and the aggregated risk ($U_{Ai}$) for all trajectory alternatives. The first calculations that are necessary here are grasped by Eqs. 3 and 4:^4^

3 $$E_{Ai} = \sum \left| {vr_{Ai,Ti} - vr_{Ai,Tj} } \right|$$

4 $$U_{Ai} = \sum vr_{Ai,Ti}$$

The degree to which the risk distribution principles $E_{Ai}$ and $U_{Ai}$ are factored in and are decisive for the selection of the final trajectory is predetermined with a weighting factor for each principle (i.e., $w_{E}$ and $w_{U}$). Exemplary weighting factors are provided in Table 7.

**Table 7:** Overview of calculated risk inequality (${\text{E}}_{{{\text{Ai}}}}$) and aggregated risk (${\text{U}}_{{{\text{Ai}}}}$) for all remaining trajectory alternatives

Trajectory alternatives ($A_{i}$)	Traffic participants ($T_{i}$)				Risk distribution principles	
	$T_{1}$	$T_{2}$	$T_{3}$	$T_{4}$	$E_{Ai}$	$U_{Ai}$
	$vr_{Ai,T1}$	$vr_{Ai,T2}$	$vr_{Ai,T3}$	$vr_{Ai,T4}$		
$A_{1}$	0.13	0.12	0.00	0.00	0.51	0.25
A_2_	0.18	0.01	0.13	0.00	0.66	0.32
$A_{4}$	0.27	0.00	0.00	0.04	0.85	0.31

Given these weightings, the SDV can select the final action, i.e., the trajectory with the lowest principle-weighted risk ($wr_{Ai}$). The corresponding calculation is grasped by Eq. 5:

5 $$wr_{Ai} = w_{E} E_{Ai} + w_{U} U_{Ai}$$

***Exemplary elaboration****. *Table 7* illustrates the calculated risk inequality and aggregated risk for each remaining trajectory alternative. In the imagined situation, the first trajectory alternative (*$A_{1}$*) satisfies the utilitarian principle most because it offers the lowest aggregated risk (*$E_{A1}$ = *0.25). Furthermore, this trajectory alternative performs best concerning the Equality principle as it entails the lowest risk inequality (*$U_{A1}$ = *0.51) between all parties involved.*

*As a next step, the SDV has to calculate the principle-weighted risk figures by drawing on the predetermined weighting factors (as illustrated in *Table 6*). As an example, the weighting factors for both – risk inequality (*$w_{E}$*) and the aggregated risk (*$w_{U}$*) – are here both 0.5.*^5^* Therefore, as illustrated in *Table 8*, the SDV will conclude that* $A_{1}$ *will entail the best outcome since this trajectory results in the lowest principle-weighted risk figure (*$wr_{A1}$ = *0.38). Ultimately, the SDV will select* $A_{1}$ *for action execution.*

**Table 8:** Overview of calculated principle-weighted risks ($wr_{Ai}$) for all remaining trajectory alternatives

Trajectory alternatives ($A_{i}$)	Weighted risk distribution principles		Principle-weighted risks ($wr_{Ai}$)
	$w_{E} E_{Ai}$	$w_{U} U_{Ai}$	
$A_{1}$	0.255	0.125	0.38
A_2_	0.33	0.16	0.49
$A_{4}$	0.425	0.155	0.58

## Discussion

In this section, we will highlight the benefits of the previously sketched decision-making process (“Benefits of this Ethical Decision-Making Model” section.), its limits and point to future research by listing (how to determine) the underlying terms that need concretization (“Limitations, Research Agenda and Terms to be Determined” section.).

### Benefits of this Ethical Decision-Making Model

Having in mind the previously sketched requirements for an SDV’s ethical decision-making process (see “Theoretical Fundamentals” section), our proposed model works towards generating the following benefits:

- *The proposed model provides a chronological order, consistency and justifications for particular decision-making steps while leaving room for adjustments.* This model states a precise sequence of steps an SDV could engage in and execute an ethical decision-making process (i.e., select the most appropriate trajectory). The whole decision procedure is elaborated in more detail by running the steps through an imagined traffic scenario. Since the model is derived from the theoretical fundamentals stated in “Theoretical Fundamentals” section, its basic structure aims to—as proposed by Dignum (2019)—align with existing regulations, standards, normative ethical theories and societal values and thereby derive explanatory and justificatory power (Jacobs & Huldtgren, 2021). While its basic structure of five steps is here assumed as static, the model offers many possibilities for adjustments by, for example, not putting the list or figures for duties, thresholds or weightings (e.g., $c_{max}$, $w_{E}$) in concrete numerical terms.
- *The proposed model utilizes overall risk (i.e., safety) as a key factor (starting with step 1).* In line with reality, in which the occurrence of events during traffic operations is uncertain (Liu, 2017) and in line with previously stated policymakers’ calls, this model adopts risks (i.e., the product of calculation of collision probability and estimated harm) as a key pillar from the beginning. As suggested in “Theoretical Fundamentals” section, this model showcases an exemplary process for an SDV maneuver to reach a ‘minimal risk condition’, which concentrates on safety as an ultimate aim. Furthermore, the underlying risk distribution considers not only the SDV’s passengers (e.g., as illustrated in Fig. 1, the SDV, two other vehicles and a pedestrian are considered), thereby striving for equal consideration of all involved road users as a starting point.
- *The proposed model adapts to the context at hand (e.g., in step 2).* Based on the SDV’s ability to fulfill certain duties that are linked to particular risk metrics, traffic situations are separated into a ‘hazard situation mode’ or ‘non-hazard situation mode’. This typification is decisive for the following decision-making process. For example, as suggested in “Adjustments to the Underlying Calculation Depending on the Situation’s Criticality” section, once a traffic situation is identified as critical, the proposed model relies on the single parameter of safety (i.e., physical integrity) in its subsequent calculations. Other parameters, such as passenger comfort or mobility, could be accounted for in less critical traffic situations. Therefore, this model responds to situational demands and context information to choose between ethical principles (Woodgate & Ajmeri, 2022).
- *The proposed model accounts for the reasonableness of risk impositions (e.g., in steps 3 and 5).* As stated in “Implementing a Mix of Ethically Grounded and Socially Shared Principles” section, it may not be reasonable to forego trajectories that could entail fatal harm at any price, especially when the occurrence of a collision is unlikely in the first place. Therefore, in step 3, this model aims to manifest such reasonableness of risk-taking by instantiating a ‘conditional Maximin strategy’, which means that, for example, a high level of estimated harm is only accepted if a low collision probability accompanies it. Therefore, trajectories that entail high levels of estimated harm are not per se to be rejected. The notion of reasonableness is further addressed in step 5, in which different ethical theories (such as the Equality principle and utilitarianism) are weighted to—in line with suggestions made by Berkey (2022)—allow the consideration and reduction of aggregate risk within a traffic scenario.
- *The proposed model incorporates responsibility considerations and the protection of vulnerable road users (e.g., in step 4).* In step 4 of this model, traffic participants are categorized into road user types in line with their relative vulnerability. Similar to existing propositions (see “Responsibility Considerations and the Protection of Vulnerable Road Users” section.), road user types include vehicles, cyclists and pedestrians. For these three types, corresponding valence factors are allocated, whereby pedestrians receive the highest and vehicles receive the lowest valence factor. Therefore, this step ensures special and double^6^ protection of vulnerable traffic participants and the incorporation of responsibility because the SDV algorithm acknowledges and compensates for the varying levels of risk that different parties introduce to traffic in the first place. Namely, the selected trajectory ($A_{1}$) warrants a high distance between the SDV and the existing vulnerable traffic participant ($T_{4}$).
- *The proposed model relies on a mix of ethically grounded and socially shared principles (e.g., in step 5).* Although this approach can be considered structurally consequentialist (Evans, 2021), a plurality of (ethical) theories make up the body of the decision-making process, namely, risk management in the form of outcome calculations and thresholds, deontological ethics in the form of duties, the Maximin principle in the form of a constraint, utilitarianism (i.e., by minimizing aggregated risk) or the Equality principle (i.e., by reducing risk inequality) in the form of distribution strategies that are weighted against each other. In addition, with the many figures (e.g., $c_{max}$, $v_{i}$, $w_{E}$) being left open to be fixated, this model leaves space for the alignment with societal values and preferences (e.g., about unacceptable risk thresholds, desired hierarchical order of road user types or weights for particular risk distribution strategies). By generally providing opportunities to combine ethical theories with insights from descriptive ethics, this model aims to forgo “the risk of attending to a set of values that is unprincipled or unbounded” (Jacobs & Huldtgren, 2021, p. 23).

### Limitations, Research Agenda and Terms to be Determined

**Fig. 2:**
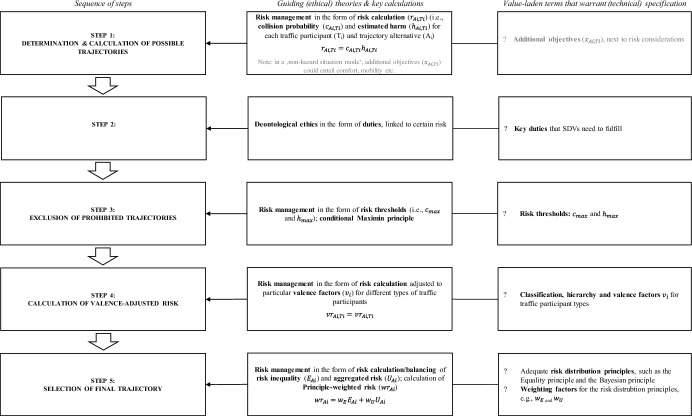
Proposed model summarizing the decision-making steps, guiding theories, underlying calculations and terms that warrant (technical) specification

Despite the previously sketched benefits, this proposed decision-making model has limitations. The following issues warrant careful consideration and demand further investigation:

- *Potential technical issues, inherent biases and tradeoffs*. From a technical perspective, this model is an abstraction from reality. For example, our decision-making model assumes SDVs have the capability to identify hazardous situations and act appropriately in a prompt manner. However, ethical computation and motion planning processes might require more time (e.g., 2 ms per trajectory) than state-of-the-art algorithms that do not integrate ethical considerations (Geisslinger et al., 2023a, 2023b). This may limit the SDVs’ ability to respond in real time. Therefore, future research should investigate whether the extra time required to undergo the suggested decision-making steps is practically achievable in due time without risking a collision. Moreover, although this model explicitly refrains from discriminating traffic participants based on personal characteristics and seeks to protect vulnerable road users, inherent biases may nevertheless creep in and influence the SDV’s outcomes. For example, AI object detection systems may struggle to recognize individuals with darker skin tones (Wilson et al., 2019), LiDAR sensors can more easily detect larger objects (e.g., trucks) compared to smaller objects such as pedestrians (Zhang et al., 2020). Without the (timely) identification and inclusion of these traffic participants into the SDV’s calculations, the efficacy of our deliberately non-discriminatory decision-making model will be undermined. Lastly, while this model strives to incorporate numerous fundamental ethical principles simultaneously, it involves implicit tradeoffs since not all requirements/components are mutually compatible. To resolve these inconsistencies (e.g., equal risk distribution vs. minimization of aggregated risk), we stress the importance of including societal preferences or legal considerations as helpful ‘tie-breakers’ “to resolve fundamental ethical disagreements, and thus garner public acceptability” (Evans, 2021, p. 324).
- *Determining numerical figures and technical measures for value-laden terms*. As illustrated in Fig. 2, a few terms^7^ that underlie each step of the proposed model are stated—on purpose–in an abstract manner at this stage. To turn this proposed model into practice, these terms will need to be concretized as numerical figures or derived from technical indicators in the future. These terms include precisely the fixation of *duties,* thresholds for collision probability ($c_{max}$) and estimated harm ($h_{max}$), the classification, hierarchy and valence for certain traffic participant types ($v_{i}$), relevant *risk distribution principles* and their corresponding weighting factors (e.g., $w_{E}$ and $w_{U}$). To approximate the numerical figures of some of these terms, the *technical measures/indicators in *Table 9 may serve as a starting point. For example, to ensure compliance with duties such as “The lives of traffic participants must not be put in harm’s way” (Evans et al., 2020), these duties will need to be attached to specific risk figures (as illustrated exemplarily in Table 2), which, in turn, can be based on indicators such as time-to-collision or time-to-react (Wishart et al., 2020). In addition, the value-laden terms can draw on *established measures in other fields*. For example, utilized principles in other fields (e.g., in healthcare: treating people equally vs. maximizing total benefits) (Persad et al., 2009) can point to appropriate risk distribution principles and their relative importance (i.e., weighting). When it comes to radiation exposure, Hansson (2007) proposes the combination of individual dose limits and collective dose levels, where the former should be given priority. Similarly, the determination of the two different threshold figures (i.e., $c_{max} , h_{max}$) could be based on established thresholds in other fields, such as individual dose limits of radiation exposure (Goodall, 2016). Furthermore, numerical figures of the value-laden terms could be approximated through *deliberations among experts* and informed by indicated preferences of other key stakeholders, such as the *broader society* (Poszler et al., 2024). For example, experts of Germany’s national ethics committee for automated and connected driving prohibited factoring in road users’ personal characteristics in SDV calculations (Lütge, 2017). This has implications for, amongst others, the ultimate determination of traffic participant types in that, for example, age shall not be a technical measure/indicator for this classification. The broader society can contribute to establishing numerical figures for the value-laden terms by indicating their preferences in empirical studies. For example, Meder et al. (2019) asked participants to state the minimum likelihood of colliding with a pedestrian (similar to $c_{max}$) in order to grant lane departure to an SDV in a dilemma situation. Similarly, to supplement our model, future research could ask participants to decide between various traffic scenarios that implicitly reflect different risk distributions (e.g., Equality principle and Bayesian principle), thereby revealing the relative importance of particular distribution principles.
- *Empirical evaluations of the ethical decision-making process as a next step.* Assessing the actual impact, effectiveness and admissibility of implementing the ethical principles and the decision-making process outlined here requires an empirical evaluation and testing of the entire model. Before real-world deployment, simulations could shed some light on the feasibility of implementing particular ethical theories, potential arising tradeoffs or destructive outcomes (Hoffmann, 2021), for example, regarding fairness or safety levels (Eastman et al., 2023). These evaluations would, amongst others, test the degree to which the algorithm performs as intended or if key requirements are ultimately undermined and measure societal and individual repercussions (Awad et al., 2022), such as the level of traffic flow efficiency or the number of traffic-related casualties. To address one of the earlier mentioned potential technical issues, such simulations could also assess whether adequate time is available for computing ethical parameters in particular traffic situations (UNECE, 2021). Additionally, it could be investigated how particular SDVs’ ethical decision-making processes function (or represent an improvement for traffic participants) in comparison to motion planning frameworks that car manufacturers actually use. Geisslinger et al. (2023a) can serve as an example of a simulation showing how risk distributions among traffic participants change when particular ethical theories are implemented into an SDV’s trajectory planning algorithm. Similarly, scholars and practitioners could validate the values and decision-making model outlined here through corresponding simulations in the future.

**Table 9:** Definition and technical specification of key terms for the SDVs’ decision steps

Terms	Definitions	Technical measures/indicators (Examples & further readings^a^)
Trajectory alternatives (A)	Set of possible actions/trajectories (physically) available to an SDV (e.g., Evans, 2021; Geisslinger et al., 2023b)	Driveable area (Lin & Althoff, 2023) Potential fields for crossable and non-crossable obstacles and road boundaries (Wang et al., 2020) Reachable sets (Coskun, 2021)
($A_{i}$)	Trajectory *i*	
Traffic participants (T)	Set of all traffic participants in a scenario	
($T_{i}$)	Traffic participant *i*	
Risk ($r_{Ai,Ti}$)	Combination of the probability of occurrence of harm and the severity of that harm (e.g., ISO, 2018b)	Product of collision probability and estimated harm (D’Souza et al., 2023; Geisslinger et al., 2021; Geisslinger et al., 2023a/b; Li et al.,; 2024; Mustafa et al., 2024; Trauth et al., 2023; Trauth et al., 2024) Pedestrian risk index (Westhofen et al., 2023)
Collision probability ($c_{Ai,Ti}$)	Likelihood of a collision happening	Spatio-temporal overlap, lateral and longitudinal separation distance between two vehicles (D’Souza et al., 2023; Mori, 2024; Mustafa et al., 2024; Wang et al., 2024) Time-to-reach (Aksjonov & Kyrki, 2023); Time-to-collision; Time-to-react (Abdelhalim & Abbas, 2022; Geisslinger et al., 2023b; Lin & Althoff, 2023; Mu et al., 2024; Westhofen et al., 2023; Wishart et al., 2020; Zhou et al., 2024) Crash potential index; aggregated crash index (Lin & Althoff, 2023; Westhofen et al., 2023) Proximity to the predicted trajectory of other traffic participants (Evans, 2021)
Estimated harm ($h_{Ai,Ti}$)	Severity of a collision in terms of the damage to the physical integrity of a human being (e.g., Evans, 2021; Geisslinger et al., 2021)	Delta-v—change in velocities following the collision (Evans, 2021; Evans et al., 2023; Lin & Althoff, 2023; Robinson et al., 2021; Zhou et al., 2024) Velocities and masses of colliding traffic participants (Evans, 2021; Geisslinger et al., 2023a; Mustafa et al., 2024; Robinson et al., 2021; Trauth et al., 2023; Vakili et al., 2024; Westhofen et al., 2023) Impact areas and/or angles (Evans, 2021; Geisslinger et al., 2023a; Mustafa et al., 2024; Trauth et al., 2023) Potential crash severity index (PCSI) determined by factors such as characteristics of the obstacles, approaching velocity, relative crash angle and weight difference (Wang et al., 2020) Collision harm index determined by velocities and masses of colliding traffic participants as well as these corresponding SDV properties post-collision (D’Souza et al., 2023) Risk of fatality; Maximum Abbreviated Injury Score (MAIS) (Evans et al., 2023; Geisslinger et al., 2023a; Robinson et al., 2021; Trauth et al., 2023) HIC_15_ head injury criterion/AIS3 + (Guo et al., 2024) KABCO injury classification scale (Wishart et al., 2020)
Additional utilities/objectives ($x_{Ai,Ti}$)	Outcome figures for SDVs’ desirable goals, next to safety	Comfort or mobility (determined by, for example, the vehicle’s acceleration and jerk) (2023b; Geisslinger et al., 2023a; Samiuddin et al., 2024) Efficiency (determined by velocity or travel time), comfort (determined by longitudinal and lateral acceleration) (Aksjonov & Kyrki, 2023; Guo et al., 2024; Sánchez et al., 2024) Cost function that integrates objectives such as comfort or energy consumption (Németh, 2023) Path tracking or occupant comfort (determined by change in steering of lateral front tire force) (Thornton et al., 2016)
Maximum acceptable collision probability ($c_{max}$)	Threshold for collision probability that must not be exceeded, given a particular estimated harm figure is exceeded	
Maximum acceptable estimated harm ($h_{max}$)	Threshold for estimated harm that must not be exceeded, given a particular collision probability figure is exceeded	Current safety levels with human drivers in conventional cars (Geisslinger et al., 2023a) MAIS3 + level injury (Evans et al., 2023)
Traffic participant valence ($$v_{i} )$$	Weighting factor for traffic participant *i* according to their relative vulnerability	
Valence-adjusted risk ($vr_{Ai,Ti}$)	Risk figure adjusted by the particular valence of traffic participant *i*	
Risk inequality ($E_{Ai}$)	Sum of risk differences among all traffic participants in trajectory *i*	Equality principle (Geisslinger et al., 2021, 2023a); egalitarian approach (Evans et al., 2023)
Aggregated risk ($U_{Ai}$)	Sum of expected harms for all traffic participants in trajectory *i* (e.g., Evans et al., 2023)	Bayesian principle (Geisslinger et al., 2021, 2023a); utilitarian approach (Evans et al., 2023)
Risk inequality weighting ($w_{E}$)	Weighting factor for the distribution principle relating to risk inequality	
Aggregated risk weighting ($w_{U}$)	Weighting factor for the distribution principle relating to aggregated risk	
Principle-weighted risk ($wr_{Ai}$)	Risk figure that balances principles related to risk inequality and aggregated risk according to their particular weighting factors	Risk-cost function that balances different ethical principles (Geisslinger et al., 2021, 2023a; Li et al., 2024)

To pinpoint relevant readings, the structured literature review by Poszler et al. (2023) served as a starting point. Specifically, we have included the ten publications identified in this review, which discuss ‘elaborate decision processes’ (e.g., ethical trajectory-planning algorithms) for SDVs, along with supplementary readings that cited these ten publications. The technical measures/indicators proposed in these publications may represent an improvement to existing patents that propose fixed risk magnitudes or collision probabilities for, for example, the incident of “hitting a pedestrian who runs into the middle of the road” (e.g., Teller & Lombrozo, 2023, p. 10)

## Conclusion

Overall, this paper aims to establish an ethical decision-making process for SDVs in hazardous situations. In particular, expanding on existing approaches (e.g., Evans et al., 2020; Poszler et al., 2023; Robinson et al., 2021), this proposed model states where exactly which (ethical) theories and requirements may apply during the decision-making process and how they could be represented (as numerical figures) in an SDV’s calculation. Although not exhaustive and resolute, this approach highlights some key considerations indicated by policymakers, standardization organizations and scholars at this moment in time. Namely, the model utilizes overall risk (i.e., safety) as a central factor and takes into account the context, reasonableness and responsibility considerations as well as the protection of vulnerable road users. Furthermore, the model allows the integration of a mix of ethical theories and societal values and overall, provides a chronological order for particular decision-making steps while leaving room for future adjustments. The requirements and technical specifications provided in this article can serve as a register for contemporary SDV developers, manufacturers or so-called ‘value leads’ when eliciting relevant ethical considerations and turning them into concrete system features. Like any other proposed decision-making process (e.g., Evans et al., 2023), our model will require ongoing evaluation and adaptation to address emerging legal restrictions, ethical standards or technical advancements.

## Appendix

**Table:** 

Number of standard	Title	Date of publication/update
ETSI TS 101 539	Intersection collision risk warning	June 2018
ISO 22078:2020	Intelligent transport systems—Bicyclist detection and collision mitigation systems (BDCMS)—Performance requirements and test procedures	February 2020
ETSI TS 103 300–2 V2.2.1	Intelligent Transport Systems (ITS); Vulnerable Road Users (VRU) awareness; Part 2: Functional Architecture and Requirements definition; Release 2	April 2021
ISO 21448:2022	Road vehicles—Safety of the intended functionality	June 2022
ISO 19638:2018	Intelligent transport systems—Road boundary departure prevention systems (RBDPS)—Performance requirements and test procedures	September 2018
ISO 26262–1	Road vehicles—Functional safety—Part 1: Vocabulary	December 2018
SAE_J3016	Taxonomy and Definitions for Terms Related to Driving Automation Systems for On-Road Motor Vehicles	April 2021
SAE J3206	Taxonomy and Definition of Safety Principles for Automated Driving System (ADS)	July 2021
IEEE P2846	P2846—Assumptions for Models in Safety-Related Automated Vehicle Behavior	March 2022
ISO 23375:2023	Intelligent transport systems—Collision evasive lateral manoeuvre systems (CELM)—Performance requirements and test procedures	February 2023
ISO/TR 4804:2020	Road vehicles—Safety and cybersecurity for automated driving systems: Design, verification and validation	December 2020
ISO 39003	Road traffic safety (RTS)—Guidance on ethical considerations relating to safety for autonomous vehicles	July 2023

Country/issuer	Title of policy document/regulation	Date of publication/update
Germany—Federal Office of Justice	Road Traffic Act (Straßenverkehrsgesetz)	November 2023
USA—U.S. Department of Transportation & National Highway Traffic Safety Administration	Federal Automated Vehicles Policy: Accelerating the Next Revolution in Roadway Safety	September, 2016
USA, California—California Department of Motor Vehicles	Article 3.7. Testing of Autonomous Vehicles	April 2022
USA, Nevada—Nevada Legislature	NV Rev Stat § 482A.044 (2022)	May 2022
USA—National Association of City Transportation Officials	Nacto Policy Statement on automated vehicles	June 2016
USA—National Highway Traffic Safety Administration	Occupant Protection for Vehicles With Automated Driving Systems	March 2022
European Commission—Directorate-General for Research and Innovation	Ethics of Connected and Automated Vehicles: Recommendations on road safety, privacy, fairness, explainability and responsibility	June 2020
European Commission	EUR-Lex—32022R1426: Uniform procedures and technical specifications for the type-approval of the automated driving system (ADS) of fully automated vehicles	August 2022
United Nations Economic Commission for Europe	New Assessment/Test Method for Automated Driving (NATM) Guidelines for Validating Automated Driving System (ADS)—amendments to ECE/TRANS/WP.29/2022/58	September 2022
Canada—Transport Canada	Guidelines for testing automated driving systems in Canada	August 2021
United Kingdom—Center for Data Ethics and Innovation	Responsible Innovation in Self-Driving Vehicles	August 2022
United Kingdom—Department for Transportation	The Highway Code	September 2023
France—Ministères Écologie Énergie Territoires	Safety validation of automated road transport systems: clarification through the analysis of accident data	July 2022
Australia—National Transport Commission	The regulatory framework for automated vehicles in Australia: Policy paper	February 2022

Further lists of related standards can be found, for example, here: https://www.connectedautomateddriving.eu/standards/standards-list/

## Footnotes

1 In this paper, we will not provide an equation for calculating utilities/goals other than safety and take the numerical figure for ${x}_{Ai,Ti}$ in Table 2 as given. This is because this paper focuses on the ‘hazard mode’, in which the key objective of SDVs is safety. Please refer to the indicated references in Table 9 for further readings on calculating utilities such as mobility or comfort.

2 While the imagined traffic scenario assumes the existence of ${T}_{2}$, ${T}_{3}$, ${T}_{4}$ and the wall, these individuals/objects are not understood as certainties. This is accounted for by factoring in the collision probability, which results from uncertainties in autonomous driving, such as the actual localization of traffic participants (Geisslinger et al., 2023a).

3 The further course of action for the ‘non-hazard mode’ is not further elaborated here since the focus of this article is SDVs’ decision-making in hazardous situations. Potential steps in the ‘non-hazard mode’ include balancing all consequences such as safety, comfort and mobility.

4 The risk disparities across all combinations of traffic participants will be summed up to determine the risk inequality for a particular trajectory alternative.

5 If other weighting factors were utilized here, the preferred decision may diverge between Table 7 and Table 8.

6 Note: ‘Double’ protection for vulnerable road users results because, by nature, their inherent risk figures are already elevated due to their higher estimated harm. In step 4, these risk figures will be further heightened compared to those of other road users, serving as an additional buffer.

7 The focus will be on the terms that carry ethical dimensions, i.e., are value-laden (such as maximum acceptable risk thresholds). By contrast, ${c}_{Ai,Ti}$ in step 1 will not be listed here since the calculation of collision probabilities is primarily an ‘objective’, technical matter as it depends, for example, on uncertainty estimations due to occluded areas (Nolte et al., 2018) that may emerge from existing obstacles in a traffic situation (ETSI, 2021a, 2021b).
